# Chlamydia trachomatis infection among patients attending sexual and reproductive health clinics: A cross-sectional study in Bao'an District, Shenzhen, China

**DOI:** 10.1371/journal.pone.0212292

**Published:** 2019-02-19

**Authors:** Rui-Lin Yan, Yun-Feng Ye, Qin-Ying Fan, Yan-Hui Huang, Gui-Chun Wen, Li-Mei Li, Yu-Mao Cai, Tie-Jian Feng, Zhi-Ming Huang

**Affiliations:** 1 Shenzhen Baoan Center for Chronic Disease Control, Shenzhen, China; 2 Shenzhen Center for Chronic Disease Control, Shenzhen, China; University of Texas Health Science Center at San Antonio, UNITED STATES

## Abstract

This study aimed to estimate the prevalence of chlamydial trachomatis (CT) infection and explore its risk factors among patients attending sexual and reproductive health clinics in Shenzhen, China. We collected demographic and clinical information from attendees (aged 18–49). CT and Neisseria gonorrhoeae (NG) infection was determined by nucleic acid amplification test (NAAT) on self-collected urine specimens. Of 1,938 participants recruited, 10.3% (95% confidence interval [CI]: 9.6%-11.0%) tested positive for CT. Prevalence was similar between men (10.6% [85/804]; 95% CI, 9.5%–11.7%) and women (10.1% [115/1134]; 95% CI: 9.2%–11.0%). Being 18–25 years old (adjusted odds ratio [aOR] = 2.52; 95%CI:1.35–4.71), never tested for CT before (aOR = 2.42; 95%CI: 1.05–5.61) and infected with NG(aOR = 3.87; 95%CI: 2.10–7.10) were independently associated with CT infection. We found that CT infection is prevalent among patients attending sexual and reproductive health clinics in Shenzhen, China. A comprehensive program including CT screening, surveillance and treatment is urgently needed.

## Introduction

Chlamydia trachomatis (CT) infection is one of the most common bacterial infections in the world, with about 73.7 million new cases occurred globally in 2015 [1]. Although infection may be asymptomatic in more than 80% of cases [2,3], 16% of cases may suffer from clinical pelvic inflammatory disease (PID), which may result in future infertility or ectopic pregnancy [4]. A population-based study in Canada has found that compared to those who tested negative for CT infection, those tested positive have a 55% increased risk of PID [5]. Another study also reported that an estimated 20% of PID, 5% of ectopic pregnancy and 29%-45% of tubal factor infertility were attributed to CT infection [6]. The risk of reproductive tract morbidity increases with repeated CT infection [5,7,8], which also increases the risk of preterm delivery in pregnant women[9], as well as epididymo-orchitis and infertility in men [10,11]. Because of its asymptomatic features [2,3] and unavailability of effective vaccines [12], CT infection control mainly relies on screening followed by case management. The diagnosis and treatment of asymptomatic CT infection through CT screening not only reduce infection duration and related complications, but also decrease latent transmission between partners and thus decelerate its spread among the population. CT screening has been widely proven to be a cost-effective method for disease control and management, particularly in high-risk populations such as sexual and reproductive health clinic attendees[13–15]. Hospital data from several countries also demonstrated declining trends in PID and ectopic pregnancy during periods of increasing CT testing and diagnosis [16–19].

Several high-income countries including Europe [20], Australia [21], Canada [22], UK [23] and the USA [24,25] recommend yearly opportunistic CT screening for all sexually active women or both women and men under 25-years-old or other population at risk. As the most populous country in the world, China plays an important role in global effort of CT infection control to decrease disease burden. As reported in previous studies, the prevalence of CT infection was as high as 13.2%-58.6% in female sex workers [26–30] and 24% in men who have sex with men (MSM) [31]. However, population-based active CT screening is still lacking in China, and the diagnosis of CT infection still relies on clinic-based passive testing on patients attending sexual and reproductive health clinics, who usually come after symptoms emerged. The current symptom-oriented testing has led to a large numbers of asymptomatic patients undetected, which poses a great threat to CT infection control. An increasing number of studies on CT infection have been conducted among female sex workers [26–30], MSM [31] and general population [32] in China, but study on CT infection among patients attending sexual and reproductive health clinics is still lacking in recent years, with only two conducted in 2009 [33] and 2005 [34].

Shenzhen, a 'special economic zone' with a land area of 1997.3 sq. km, is located in the south coastal China. With a GDP (gross domestic product) of 2.2 trillion (RMB), Shenzhen ranks the third among all cites of mainland China [35]. There are 12.5 million permanent residents in total with a median age of 31 years old, 65.3% of who are non-registered [35]. Along with the rapid economic growth and increasing sexually active migrant population, Shenzhen has witnessed an alarmingly rapid spread of sexually transmitted diseases (STDs) in recent years. The incidence of CT infection increased from 137.88 per 100 000 in 2008 to 189.57 per 100 000 in 2016, with an average annual growth rate of 4.06% [36], much higher than other cities of China [37]. The reported incidence rate was the highest among women aged 25-34-years old—more than 500.00 per 100 000 [36]. A population-based study in 2018 also reported that CT infection was prevalent among women with a prevalence of 4.12% [32]. In response to the pressing need of CT infection control and reproductive health improvement, the Health and Family Planning Commission of Shenzhen Municipality launched a pilot project called Shenzhen Gonorrhea and Chlamydia intervention pilot (SGCIP) in 2017 to expand the active screening of CT among patients attending sexual and reproductive health clinics. The current study aims to report the prevalence and risk factors of CT infection in a baseline survey, to provide guidance for future integrated interventions to reduce the burden of CT infection in China.

## Materials and methods

### Recruitment of participants

The survey was conducted at the Bao'an District of Shenzhen City during April and May 2018. Bao'an District is located in the west of Shenzhen City with the largest area and biggest population among all of the ten districts. A total of 12 hospitals located in six sub-districts of Bao'an District provide CT infection diagnosis service for patients. Ten clinics, including three STD clinics, four gynaecology clinics and three genitourinary clinics, were selected from the 12 hospitals based on patient flow (measured by the number of newly diagnosed CT cases in 2017). STD clinics refer to clinics specially set up for both male and female attendees with STD symptoms or high risk sexual behaviors or have sexual partners with STD. Genitourinary clinics refer to clinics set up for the male attendees with any genitourinary symptoms, including symptoms related to STD. Gynaecology clinics refer to clinics set up for female attendees with any needs in sexual and reproductive health care, including STD. Attendees presenting to the sexual and reproductive health clinics who were 18–49 years old and sexually experienced were invited to participate in the study. Exclusion criteria includes having taken antibiotics in the preceding 28 days and unwilling to take a urine specimen.

The Ethical Review Committee of the Bao'an Center for Chronic Disease Control reviewed and approved the study. All eligible attendees were informed of the purpose and details of the study including information and specimen collection by a doctor. Participants' information and laboratory testing results were recorded anonymously and were only used for research purpose without being disclosed. Participants who tested positive for CT and/or NG were contacted privately by the initial doctor for further treatment and other interventions. Attendees have the right to decide whether to join the study and for those who joined, a written informed consent was signed by each participant before the study.

The sample size was calculated based on an estimated CT infection prevalence of 10.0% [38], α of 0.05 and precision of 15%, leading to a sample size of 1537. Assuming a refusal and/or non-evaluable rate of approximately 15.0%, we expanded our sample size to 1808 and recruited 1900 clinic attendees in the final study.

### Information collection

Participants were interviewed in a private setting by the doctor to complete a set of anonymous structured questionnaire and provide self-collected urine specimen. The questionnaire included information on demographic characteristics (including age, education and marital status, etc.), history of CT test and diagnosis, sexual orientation and sexual behavior (extramarital sex), presence of symptoms suggestive of a bacterial sexually transmitted infection as checked and recorded by the doctor (vaginal discharge, cervical hyperemia and discharge, dysuria and urethral discharge, and lower abdominal pain in females; dysuria and urethral discharge, pain and swelling of one or both testicles in males). 3–5 ml of first-catch urine specimen was collected using the Cobas urine specimen collection kit (Roche P/N 05170486190).

### CT testing

All specimens were temporarily stored at 4°C in the local laboratory for a maximum of 24 hours before being transported to a central laboratory for testing. The urine specimens were assayed for CT and NG based on polymerase chain reaction (PCR) of the Cobas 4800 System (Roche, Switzerland) according to the manufacturer's instructions.

### Clinical management

For patients who tested positive for CT or NG, clinical management involving treatment, follow-up visits and health education were provided according to the national guidelines [39].

### Statistical analysis

All data from questionnaire and laboratory tests were entered into Epidata database by two investigators with data consistency checked by a third person. Chlamydia prevalence among patients attending sexual and reproductive health clinics was estimated as the proportion of those with positive test results among those tested, with 95% confidence intervals. Factors associated with CT infection were also investigated with unadjusted odds ratios (ORs), adjusted odds ratios (aORs) and 95% confidence intervals calculated using a random-effects logistic regression model. A P values of p<0.05 were considered statistically significant. All statistical analyses were conducted using SPSS Statistics for Windows Version 17.0.

## Results

### Recruitment of participants

Among the 1963 participants, 1938 (98.7%) completed both the questionnaire survey and urine specimen test and were included for final analyses. Of the 1938 participants, 23.0% (445) were from STD clinics, 55.4% (1074) from gynaecology clinics and 21.6% (419) from genitourinary clinics.

### Participant characteristics

Characteristics of study participants are presented in Table 1. The average age of participants was 31.2 with a standard deviation of 7.1 (range: 18–49). Over half (58.5%) were females. A total of 1897 (97.9%) reported sexual orientation of heterosexuality and 665 (34.3%) reported extramarital sex in the past three months. 95(4.9%) reported a previous diagnosis of CT and 134 (6.9%) reported having ever tested for CT. 1015 (52.4%) reported at least one current symptom suggestive of a bacterial STI.

**Table 1:** Characteristics of participants.

Characteristics	n	%
**Age (years)**		
≤ 25	431	22.2
26–30	572	29.5
31–35	462	23.8
36–40	251	13.0
>40	222	11.5
**Sex**		
Male	804	41.5
Female	1134	58.5
**Marital status**		
Married	1346	69.5
Single	551	28.4
Divorced	41	2.1
**Clinics**		
Gynaecology	1074	55.4
Genitourinary	419	21.6
STD	445	23.0
**Census register**		
Shenzhen	280	14.4
Others	1658	85.6
**Time lived in Shenzhen (years)**		
< 2	511	26.4
≥ 2	1427	73.6
**Occupations**		
Worker	825	42.6
Server	354	18.3
Office clerk	335	17.3
Housewife	169	8.7
Individual operator	70	3.6
Others	109	5.6
Unemployment	76	3.9
**Education**		
Secondary school or below	826	42.6
Senior high school	609	31.4
College or above	503	26.0
**Medical insurance**		
Yes	1062	54.8
No	876	45.2
**Previous CT test**		
Yes	134	6.9
No	1804	93.1
**Previous CT diagnosis**		
Yes	95	4.9
No	1843	95.1
**Monthly income (Chinese Yuan)**		
<4000	459	23.7
4000–7999	1135	58.6
≥ 8000	304	15.7
Unknown	40	2.1
Monthly income of partner*(Chinese Yuan)		
<4000	280	14.4
4000–7999	719	37.1
≥ 8000	421	21.7
Unknown	518	26.7
**Sexual orientation**		
Heterosexual	1897	97.9
Homosexual or Bisexual	20	1.0
Unknown	21	1.1
**Extramarital sex in the past 3 months**		
Yes	1273	65.7
No	665	34.3
**Current symptoms of bacterial STI**		
Yes	1015	52.4
No	923	47.6
**Infected with NG**		
Yes	54	2.8
No	1884	97.2

*Partner includes legal spouse in marriage and boyfriend/girlfriend for the singles.

### Prevalence of CT

Among the 1938 participants, 200 were tested positive for CT infection, with a prevalence of 10.3% (95% CI: 9.6%-11.0%). In total, 54 participants were infected with NG, and 17 (31.5%) of them were co-infected with CT. Of the patients infected with CT, 40.5% were asymptomatic and 97.0% were never tested for CT previously. In univariate analyses (Table 2), 18–25 years old (OR = 2.75; 95%CI: 1.48–5.12; *P* = 0.001) or 26-30-years old (OR = 1.88; 95%CI: 1.01–3.51; *P* = 0.046), being single (OR = 1.61; 95%CI: 1.19–2.20; P = 0.002) or divorced (OR = 2.52; 95%CI: 1.14–5.59; P = 0.023), never tested for CT before (OR = 2.57; 95%CI: 1.12–5.91; P = 0.026), higher monthly income of partner (OR = 0.50; 95%CI: 0.30–0.84; P = 0.009), current symptoms suggestive of bacterial STI (OR = 1.38; 95%CI: 1.03–1.86; P = 0.034) and infected with NG (OR = 4.27; 95%CI: 2.36–7.74; P<0.001) were all significantly associated with CT infection.

**Table 2:** Prevalence of CT and unadjusted OR by characteristics.

Characteristics	n	CT cases	Prevalence (95%CI)	Unadjusted OR (95%CI)	*P*
**Age (years)**					
≤ 25	431	63	14.6 (12.9–16.3)	2.75 (1.48–5.12)	0.001
26–30	572	60	10.5 (9.22–11.8)	1.88 (1.01–3.51)	0.046
31–35	462	45	9.7 (8.32–11.1)	1.74 (0.92–3.29)	0.091
36–40	251	19	7.6 (5.93–9.27)	1.32 (0.64–2.73)	0.460
>40	222	13	5.9 (4.32–7.48)	1.00	-
**Sex**					
Male	804	85	10.6 (9.5–11.7)	1.00	-
Female	1134	115	10.1 (9.2–11.0)	0.96 (0.71–1.28)	0.759
**Marital status**					
Married	1346	118	8.8 (8.03–9.57)	1.00	-
Single	551	74	13.4 (11.9–14.9)	1.61 (1.19–2.20)	0.002
Divorced	41	8	19.5 (13.3–25.7)	2.52 (1.14–5.59)	0.023
**Clinic**					
Gynaecology	1074	104	9.7 (8.80–10.6)	0.93 (0.65–1.34)	0.698
Genitourinary	419	50	11.9 (10.3–13.5)	1.18 (0.77–1.80)	0.456
STD	445	46	10.3 (8.86–11.7)	1.00	-
**Census register**					
Shenzhen	280	21	7.5 (5.93–9.07)	1.00	-
Others	1658	179	10.8 (10.0–11.6)	1.49 (0.93–2.39)	0.096
**Time lived in Shenzhen (years)**					
< 2	511	62	12.1 (10.7–13.5)	1.00	-
≥ 2	1427	138	9.7 (8.92–10.5)	0.78 (0.56–1.07)	0.117
**Occupation**					
Worker	825	90	10.9 (9.8–12.0)	0.81 (0.40–1.63)	0.551
Server	354	36	10.2 (8.6–11.8)	0.75 (0.35–1.58)	0.446
Office clerk	335	33	9.9 (8.3–11.5)	0.72 (0.34–1.54)	0.397
Housewife	169	11	6.5 (4.6–8.4)	0.46 (0.19–1.13)	0.092
Individual operator	70	8	11.4 (7.6–15.2)	0.85 (0.32–2.30)	0.751
Others	109	12	11.0 (8.0–14.0)	0.82 (0.33–2.00)	0.657
Unemployment	76	10	13.2 (9.3–17.1)	1.00	-
**Education**					
Secondary school or below	826	95	11.5 (10.4–12.6)	1.00	-
Senior high school	609	63	10.3 (9.1–11.5)	0.89 (0.63–1.24)	0.489
College or above	503	42	8.3 (7.1–9.5)	0.70 (0.48–1.03)	0.068
**Medical insurance**					
Yes	1062	98	9.2 (8.3–10.1)	1.00	-
No	876	102	11.6 (10.5–12.7)	1.30 (0.97–1.74)	0.082
**Previous CT test**					
Yes	134	6	4.5 (2.7–6.3)	1.00	-
No	1804	194	10.8 (10.1–11.5)	2.57 (1.12–5.91)	0.026
**Previous CT diagnosis**					
Yes	95	9	9.5 (6.5–12.5)	1.00	-
No	1843	191	10.4 (9.7–11.1)	1.11 (0.55–2.23)	0.781
**Monthly income (Chinese Yuan)**					
<4000	459	45	9.8 (8.4–11.2)	1.00	-
4000–7999	1135	124	10.9 (10.0–11.8)	1.13 (0.79–1.62)	0.510
≥ 8000	304	26	8.6 (7.00–10.2)	0.86 (0.52–1.43)	0.561
Unknown	40	51	12.5 (7.3–17.7)	1.31 (0.49–3.52)	0.587
Monthly income of partner*(RMB Yuan)					
<4000	280	35	12.5 (10.5–14.5)	1.00	-
4000–7999	719	79	11.0 (9.8–12.2)	0.86 (0.57–1.32)	0.500
≥ 8000	421	28	6.7 (5.5–7.9)	0.50 (0.30–0.84)	0.009
Unknown	518	58	11.2 (9.8–12.6)	0.88 (0.56–1.38)	0.584
**Sexual orientation**					
Heterosexual	1897	194	10.2 (9.5–10.9)	1.00	-
Homosexual or Bisexual	20	3	15.0 (7.0–23.0)	1.55 (0.45–5.33)	0.488
Unknown	21	3	14.3 (6.7–21.9)	1.46 (0.43–5.01)	0.545
**Extramarital sex in the past 3 months**					
Yes	1273	122	9.6 (8.8–10.4)	1.00	-
No	665	78	11.7 (10.5–12.9)	1.25 (0.93–1.69)	0.141
**Current symptoms of bacterial STI**					
Yes	1015	119	11.7 (10.7–12.7)	1.38 (1.03–1.86)	0.034
No	923	81	8.8 (7.9–9.7)	1.00	-
**Infected with NG**					
Yes	54	17	31.5 (25.2–37.8)	4.27 (2.36–7.74)	0.000
No	1884	183	9.7 (9.0–10.4)	1.00	-

*Partner includes legal spouse in marriage and boyfriend/girlfriend for the singles.

### Findings from the multiple logistic regression analysis

In multivariate analyses (Table 3), participants being 18–25 years old (aOR = 2.52; 95%CI: 1.35–4.71; p = 0.004), never tested for CT before (aOR = 2.42; 95%CI: 1.05–5.61; p = 0.039) and infected with NG (aOR = 3.87; 95%CI: 2.10–7.10; p<0.001) were independently significantly associated with CT infection.

**Table 3:** Factors associated with CT: Multivariate logistic regression analyses.

Variables	Beta	Standard error	Wald *χ*^*2*^	Adjusted OR	95% CI	*P*
**Age (years)**						
≤ 25	0.92	0.32	8.35	2.52	1.35–4.71	0.004
26–30	0.58	0.32	3.28	1.78	0.95–3.32	0.070
31–35	0.53	0.33	2.58	1.69	0.89–3.22	0.108
36–40	0.27	0.38	0.50	1.31	0.63–2.72	0.478
>40						
**Previous CT test**					
Yes						
No	0.89	0.43	4.28	2.42	1.05–5.61	0.039
**Current symptoms of bacterial STI**					
Yes	0.26	0.16	2.86	1.30	0.96–1.76	0.091
No						
**Infected with NG**					
Yes	1.35	0.31	19.00	3.87	2.10–7.10	<0.001
No						

## Discussion

Our study showed that 10.6% of men and 10.3% of women attending sexual and reproductive health clinics were tested positive for CT infection, similar to that reported in England (10.3%-10.6%) [38,40], Netherlands (8.9%-10.1%) [41], Nigeria (9.6%) [42], Danmark (11.5%) [43], Spain (12.3%) [44], Iran (12.6%) [45], and Mexico (14.2%) [46]. However, the prevalence of CT infection in the current study was higher than that reported in Australia (5.9%) [47] and lower than that reported in Ethiopia (18.9%) [48], Palestine (20.2%) [49] and Solomon Islands(20.3%) [50]. Compared to the previously reported prevalence of 17.7% in Shenzhen in 2009 [33], the current study found a much lower prevalence of CT infection, which may be related to improved access to health care for city residents. From 2009 to 2016, the number of hospitals and outpatients increased by 32.7% and 39.5% respectively in Shenzhen, while the permanent population increased only by 19.7% [35], indicating increased access and utilization of medical resource. It is likely that more people with CT infection went to the clinics and received appropriate treatment. In addition, reduction of high-risk sexual behaviors following mass health education campaigns and condom use promotion may have also played an important role in the decrease of CT prevalence. Since no data were available on sexual behaviors (such as the number of partners and the use of condoms) both in our study and in the previous similar study of Shenzhen in 2009 [33], we can only make our best guess at the causal relationship between them instead of confirming it. Other possible explanations include different distribution of risk factors among different population in different geographical regions, as well as different laboratory methods with different sensitivity and specificity, which may all contribute to the differences between our results with other studies.

Consistent with previous reports [33, 34, 47, 48, 51–53] being 18–25 years old was an independent risk factor of CT infection. In addition, being infected with NG and never tested for CT preciously were also independent risk factors of CT infection. Young people are usually more sexually active and are more likely to have multiple sexual partners than older people, which greatly increase their risk of CT infection and transmission. Our findings justified the recommended screening programs in high-income countries to screen for all sexually active women or both men and women in the age groups with highest risk of infection [22–24, 54]. Being infected with NG was the strongest independent risk factor of CT infection, which may be explained by the shared exposure risk and infection route of both NG and CT. Considering the high co-infection [38, 55, 56], the CDC guidelines recommend that all patients treated for NG should also be treated for CT [54]. Those never tested for CT was independently associated with increased risk of CT infection, which may be related to their lack of health concern and thus lack of healthcare seeking behaviors. In our study, only 6.9% of the participants reported previous testing for CT, much lower than that reported in Britain [57]. Besides, 97% of infected cases were identified in participants never tested before in the current study, indicating that a large numbers of patients would be missed due to the poor screening. Consistent with most studies nowadays [34, 46, 48–50], education and occupation were not found to be significant risk factors of CT infection in our study. In the past, the spread of STD prevention knowledge mainly relies on traditional media, such as books and poster foldouts, which were usually more accessible to people with higher education and occupation. As a result, those with lower education and occupation may have less knowledge of CT prevention and thus have higher risk of CT infection. Nowadays, along with the rapid development of information technology and popularization of internet, knowledge of STD prevention can be easily accessed through a variety of public media, including the most commonly used electronic pictures and videos by people from all levels education and occupation. As a result, the impact of education and occupation on CT infection has weakened gradually. Prevalence of CT infection was similar between women and men, highlighting the importance of including men in CT control strategies as well. In accordance with previous studies [2, 3], 40.5% of diagnosed CT cases were asymptomatic patients in our study, emphasizing that syndromic management alone was not enough in controlling CT infection [58].

SGCIP was the first program launched aiming at controlling CT infection and improving the reproductive health by expanding active CT screening and strengthening case management in China. The current study enjoys the advantage of large sample size and high response rate, which ensured sufficient power to accurately estimate the CT prevalence and risk factors in sexual and reproductive health clinic attendees. Moreover, a large number of men were also included in our study, who were equally important but were often ignored in most CT control strategies.

Our study had the following limitations: first, the study was conducted among sexual and reproductive health clinic attendees in Shenzhen, a newly developed city with masses of migrant population from inland China, generalization of the results from this study should therefore be made with caution. Second, detailed information on sexual behaviors were not collected systematically, and reporting bias may exist in sensitive questions such as sexual orientation and extramarital sex since participants were usually reluctant to divulge this information. Other factors that may influence CT infection, such as multiple sexual partners and partners' infection status, were not recorded in the current study. Future studies may benefit from adding these factors in their analyses.

In conclusion, high prevalence of CT infection in our study population has called for active screening, surveillance and treatment program among patients attending sexual and reproductive health clinics. Our findings have significant implications for local and/or national government to design evidence-based programs to reduce the burden of CT infection and improve reproductive health in China.

## Supporting information

S1 Questionnaires CT prevalence survey questionnaires in English. (DOCX). CT prevalence survey questionnaires in Chinese. (DOCX).
(ZIP)
