# Supplementary material for: Chlamydia trachomatis infection among patients attending sexual and reproductive health clinics: A cross-sectional study in Bao'an District, Shenzhen, China
Source: PLoS One. 2019 Feb 19;14(2):e0212292. doi: 10.1371/journal.pone.0212292 (PMC6380618; doi:10.1371/journal.pone.0212292)
Supplement: S1 Questionnaires — (DOCX). CT prevalence survey questionnaires in Chinese. (DOCX). (ZIP) [file pone.0212292.s001.zip › Supporting Information/Supporting Information-Survey questions in Chinese.docx]

生殖道沙眼衣原体感染患病率调查问卷

01 就诊科室：①皮肤（性病）科 ②妇科 ③泌尿外科

02 性别：①男 ②女
03 年龄： ______岁
04 婚姻状况：①未婚 ②已婚 ③离异 ④丧偶
05 户口所在地：①深圳 ②非深圳
06 在深圳居住时间：①＜3月 ② 3~6月 ③ 7~12月 ④ 13 ~24月 ⑤≥ 24月

07 职业：①工人 ②服务员 ③职员 ④个体经营者 ⑤家庭主妇 ⑥ 失业/待业 ⑦其他

08 文化程度：①初中及以下 ②高中/中专 ④大专及以上
09 是否使用医疗保险：①是 ②否
10 您既往有过生殖道沙眼衣原体感染检测经历吗？ ①有 ②没有
11 您曾经被诊断为生殖道沙眼衣原体感染吗？①有 ②没有
12 性病相关症状和体征：①有 ②没有
13 月收入（人民币）： ______元；配偶^*^月收入（人民币）： ______元
14 性取向：①异性 ②同性 ③双性

15 最近 3 个月内，您是否和配偶或男/女朋友以外的人发生过性行为：①是 ②否
注：* 配偶：包括已婚者的法定配偶以及未婚者的男/女朋友。
