# Supplementary material for: Chlamydia trachomatis infection among patients attending sexual and reproductive health clinics: A cross-sectional study in Bao'an District, Shenzhen, China
Source: PLoS One. 2019 Feb 19;14(2):e0212292. doi: 10.1371/journal.pone.0212292 (PMC6380618; doi:10.1371/journal.pone.0212292)
Supplement: S1 Questionnaires — (DOCX). CT prevalence survey questionnaires in Chinese. (DOCX). (ZIP) [file pone.0212292.s001.zip › Supporting Information/Supporting Information-Survey questions in English.docx]

**CT prevalence survey questionnaires**

01 Clinics：①STD ②Gynaecology ③Genitourinary

02 Sex：①Male ②Female
03 Age (years)： ______

04 Marital status：①Single ②Married ③Divorced ④Widowed

05 Census register：①Shenzhen ②Others

06 Time lived in Shenzhen： ①less than three months ② three to six months ③ seven to twelve months ④ thirteen to twenty four months ⑤more than twenty four months
07 Occupations：①Worker ②Server ③Office clerk ④Individual operator ⑤Housewife ⑥Unemployment ⑦Others

08 Education：①Secondary school or below ②Senior high school ③College or above

09 Use medical insurance：①Yes ②No
10 Have you ever been tested for Chlamydia trachomatis? ①Yes ②No
11 Have ever been diagnosed with Chlamydia trachomatis infection? ①Yes ②No

12 Current symptoms of bacterial sexually transmitted infection：①Yes ②No

13 Monthly income (RMB Yuan)： ______； Monthly income of partner* (RMB Yuan)： ______

14 Sexual orientation：①Heterosexual ②Homosexual ③Bisexual

15 Have you ever sexed with someone other than your spouse or boyfriend/girlfriend in the past 3 months? ①Yes ②No

*Partner includes legal spouse in marriages and regular boyfriend/girlfriend for the singles .
